# West Nile Virus Knowledge among Hispanics, San Diego County, California, USA, 2006

**DOI:** 10.3201/eid1608.100067

**Published:** 2010-08

**Authors:** Jeffrey W. Bethel, Stephen H. Waterman

**Affiliations:** East Carolina University, Greenville, North Carolina, USA (J.W. Bethel); Centers for Disease Control and Prevention, Atlanta, Georgia, USA (S.H. Waterman)

**Keywords:** West Nile Virus Knowledge among Hispanics,USA, 2006, viruses, West Nile virus, Hispanics, knowledge, attitudes, practices, public health, vector-borne infections, immigrants, viruses, letter

**To the Editor:** West Nile virus (WNV), spread by infected mosquitoes, is a serious public health threat throughout the United States and can cause life-altering and even fatal disease (*1*). In San Diego County, California, the human infection rate was 0.18 per 100,000 persons during 2003–2006 (5 cases, 1 locally acquired) and then increased to 0.52 and 1.17 per 100,000 persons in 2007 and 2008, respectively, despite few changes in surveillance activities (*2*). Community-based mosquito control programs, adoption of personal protective behavior (PPB), and education are the most effective ways to prevent human WNV infection because no specific antiviral drug treatment or vaccine exists (*1**,**3*). Although WNV-associated illness has occurred in all racial and ethnic groups, Hispanics are potentially at risk because of language and cultural barriers to obtaining information regarding WNV prevention (*4*). San Diego County Department of Environmental Health’s education and outreach efforts include airing public service announcements in English and Spanish on television and radio and posting information on a website and social networking sites.

In 2006 we administered a survey to assess knowledge, attitudes, and practices (KAPs) regarding WNV among Hispanics in San Diego County. According to the US Census Bureau, the county is home to >900,000 Hispanics, of whom ≈41% are foreign born (*5*). A multistage cluster sampling scheme was used to identify Hispanics >18 years of age in 3 county regions (northern, central, and southern) and has been described elsewhere (*6*). Interviewers went door to door and, for each selected household, asked to speak to a Hispanic member of the household >18 years of age who was the most knowledgeable person about residents’ health.

We examined KAPs regarding WNV by using 8 questions that were part of a larger survey assessing Hispanics’ KAPs regarding several health-related issues, including topics such as influenza and lead poisoning. We used 4 questions to assess knowledge, 1 to assess attitude, and 3 to assess practices or adoption of PPB. For example, we asked respondents “What precautions, if any, have you taken to protect yourself and/or your family against West Nile virus?” The interviewer then read a list of possible responses (e.g., removed areas of standing water, used insect repellent with DEET [N, N-diethyl-meta-toluamide]) from which the interviewee indicated yes or no. Multiple responses were allowed. Questions were based on those used in previous studies and in border-region focus groups and were modified according to input from local experts and pilot testing. Spanish translation and back translation were conducted separately by 2 translators.

Interviewers completed 226 surveys, which represented 53.8% of all houses approached and 69.5% of 325 households in which a person answered the door. Respondents’ mean age was 41 years (range 18–87 years), 79.2% were foreign born, 85.8% completed the survey in Spanish, and 65.9% were women. Overall, 149 (66.2%) of the 226 respondents were aware of WNV; key demographic covariates differed, including greater awareness among English speakers, respondents living in the United States >5 years, and respondents completing >12 years of education (Table). News media (e.g., television, radio, newspaper) were the most frequent sources cited (93.2%) for WNV knowledge, followed by doctor or healthcare professionals (12.2%). Of the respondents who had heard of WNV, 87.9% knew it was transmitted by infected mosquitoes. More than 75% of respondents described their level of concern regarding WNV as “not at all” or “somewhat.”

**Table:** Awareness of West Nile virus and PPB among survey respondents, San Diego County, California, USA, 2006*

Characteristic	WNV awareness, no. (%), n = 226	p value†	PPB use, no. (%), n = 149	p value†
Overall	149 (66.2)	–	62 (41.6)	–
Age, y‡§		0.026		0.188
18–29	37 (55.2)		14 (38.9)	
30–44	52 (65.0)		26 (50.0)	
45–64	40 (83.3)		17 (42.5)	
>65	20 (66.7)		5 (23.8)	
Education, y		0.048		0.926
<12	121 (64.7)		50 (41.3)	
>12	26 (81.3)		11 (42.3)	
Preferred language for interview		0.004		0.249
Spanish	120 (62.2)		47 (39.2)	
English	29 (90.6)		15 (51.7)	
Country of birth		0.158		0.104
United States	36 (76.6)		19 (54.3)	
Other	113 (63.5)		43 (37.7)	
Years in United States		0.004		0.183
<5	9 (34.6)		5 (55.6)	
>5	104 (68.4)		38 (36.2)	
Gender		0.602		0.042
M	49 (63.6)		15 (30.6)	
F	100 (67.6)		47 (47.0)	
Region¶		0.018		0.151
Northern	20 (48.8)		5 (25.0)	
Central	34 (64.2)		11 (31.4)	
Southern	95 (72.5)		46 (48.9)	

*WNV, West Nile virus; PPB, personal protective behavior. †Proportions were compared by using the χ^2^ test. ‡Awareness: 18–29 vs. 45–64, p = 0.003; 30–44 vs. 45–64, p = 0.064; 45–64 vs. >65, p = 0.089; other comparisons are nonsignificant. §Adoption of PPB: 30–44 vs. >65, p = 0.045; other comparisons are nonsignificant. ¶Awareness: northern region vs. southern region 3, p = 0.033; other comparisons are nonsignificant.

Among the 149 respondents who were aware of WNV, 62 (41.6%) adopted PPBs to protect themselves or their families; more women than men adopted PPBs (Table). The most frequent PPB cited was the removal of standing water around the home (58.1%), followed by use of repellent with DEET (48.4%), and repairing broken windows or screens (43.5%).

We found lower awareness of WNV among San Diego County Hispanics (66.2%) than previously reported for predominantly non-Hispanic populations (range 77.2%–99.0%) (*7**–**9*). One survey reported that 41% of 17 Spanish-speaking respondents were aware of WNV (*9*). We also identified women as the primary source of PPB adoption among Hispanic households and a potential target population for interventions. Previous studies examining KAPs regarding WNV included small numbers of Hispanics and thus were unable to identify this subgroup for targeted interventions.

The finding of low awareness, concern, and PPB adoption may have 2 possible explanations. First, the observations may be appropriate given the low incidence of WNV in San Diego County and Mexico. At the time the survey was conducted, only 1 locally acquired case of WNV infection among humans had been reported in San Diego County; through 2006, WNV was rarely reported among humans in Mexico (*10*). Second, the low levels of awareness, concern, and PPB adoption may simply reflect the priority of WNV prevention compared with other basic necessities and health risks among the largely immigrant survey population.

Differences in awareness, concern, and practices among Hispanics by age, education, gender, language, years living in United States, and region of San Diego County indicate that varied educational tactics are needed to inform this population. Most educational efforts for Hispanics are simple translations of material into Spanish, which are likely not sufficient to reach this heterogeneous population.
